# History of concussion and lowered heart rate variability at rest beyond symptom recovery: a systematic review and meta-analysis

**DOI:** 10.3389/fneur.2023.1285937

**Published:** 2024-01-22

**Authors:** Eric Wesolowski, Zubair Ahmed, Valentina Di Pietro

**Affiliations:** ^1^Institute of Inflammation and Ageing, University of Birmingham, Birmingham, United Kingdom; ^2^Centre for Trauma Sciences Research, University of Birmingham, Birmingham, United Kingdom

**Keywords:** HRV, heart rate variability, history of concussion, TBI, sports concussion

## Abstract

**Introduction:**

Concussion is a growing concern in worldwide sporting culture. Heart rate variability (HRV) is closely tied with autonomic nervous system (ANS) deficits that arise from a concussion. The objective of this review was to determine if a history of concussion (HOC) can impact HRV values in the time-domain in individuals at rest. This review works to add to the literature surrounding HRV testing and if it can be used to check for brain vulnerabilities beyond the recovery of concussion symptoms.

**Materials and methods:**

The systematic review was conducted using the Preferred Reporting Items for Systematic Reviews and Meta-Analysis (PRISMA) method. A computer based systematic review scanned articles dating from 1996 to June 2023 through PubMed, Cochrane Library, Google Scholar, and EMBASE databases. A risk of bias assessment was conducted using the ROBINS-E tool. The average difference in time between heartbeats (MeanNN), the standard deviation of the differences (SDNN), and the root mean squared of the successive intervals (RMSSD) were measured.

**Results:**

Six total studies were found that fit the inclusion criteria including a total of 242 participants (133 without HOC, 109 with HOC). The average age of the control group was 23.3 ± 8.2, while the average age of the history of TBI group was 25.4 ± 9.7, with no significant difference between the groups (*p* = 0.202). Four of the studies reported no significant difference in any of the three measures, while two of the studies reported significant difference for all three measures. The meta-analysis was conducted and found that MeanNN (*p* = 0.03) and RMSSD (*p* = 0.04) reached statistical significance, while SDNN did not (*p* = 0.11).

**Conclusion:**

The results of this meta-analysis showed significant difference in two of the three HRV time-domain parameters evaluated. It demonstrates that there can be lowered HRV values that expand beyond the recovery of symptoms, reflecting an extensive period of ANS susceptibility after a concussion. This may be an important variable in determining an athlete’s return to play (RTP). Lack of homogenous study populations and testing methods introduces potential for bias and confounding factors, such as gender or age. Future studies should focus on baseline tests to compare individuals to themselves rather than matched controls.

## 1 Introduction

Traumatic Brain Injury (TBI) is an extremely relevant worldwide health issue, with an increasing concern for its continuous impact on the personal, social, and economic wellbeing of the population. Although the prevalence of TBI’s can differ by region, globally around 30 million new TBI’s are sustained every year, and it is predicted that many more go unreported (1, 2). The two most common mechanisms of injury are falls and road traffic accidents (2). Various tests and scales are used to measure severity of a TBI, yet the Glasgow Coma Scale tends to be the most popular indicator of whether an injury is classified as mild, moderate, or severe. Moderate (GCS score of 9–12) and severe (GCS score of 8<) are far less common but lead to more complications and an increased mortality risk (1, 2). Mild TBI, also known as concussion, (GCS score of 13–15) is by far the most common, accounting for 80–90% of cases (1, 3). Concussion, is also a common sports injury, with an estimated 1.6–3.8 million reported cases in the United States each year (4). Sports related concussions, are a crucial aspect to consider, due to the potential risk of long-lasting effects on brain and cognitive health (1, 5, 6).

In today’s sporting culture, one that promotes strength and toughness by playing through injuries, it is essential to monitor instances of head trauma due to the severity of damage that can result if it is not taken seriously. One example of this is second impact syndrome, which refers to a life-threatening condition that occurs when a second head injury is sustained prior to the full recovery of the initial injury (3, 7). Although often resulting from concussive impacts, edema, contusions, and hematomas are separate forms of TBI, involving brain hemorrhage that may not be present in a simple concussion. In other severe instances, a harsh twisting or shaking of the brain can cause axonal injuries that can deteriorate neural connections and function. Whether it be a concussion or some other form of TBI, many collegiate and professional athletes tend to downplay any symptoms they may have, in fear of losing playing time and opportunities (8, 9). Other important aspects to consider are the intracranial pressure (ICP) and the cerebral perfusion pressure (CPP). ICP is the pressure within the cranium, and CPP is the pressure threshold that allows oxygen to enter cerebral tissue. If a head injury were to occur that results in an increase of ICP that exceeds the CPP pressure gradient, there will be no blood flow to the brain. This will quickly result in life threatening conditions and lead to neuronal death. ICP and its effects on CPP are tied in with sympathetic activity (10), thus, heart rate and changes in HRV have been shown to be predictors of ICP levels (11). Regardless of the cause, the potentially catastrophic situation created by an ignorance to a head injury highlights the importance of in-depth and accurate evaluations for sports trainers and professionals that are working with these athletes.

One physiological marker that has gained more following in the past 20 years or so is heart rate variability (HRV). HRV measures the time between heart beats, specifically the time between the R intervals, and has many other metrics that can be measured as well. These can be divided into time-domain, frequency-domain, and non-linear HRV measures. Time-domain interval metrics look at the variability in measurements in period between successive heartbeats, while frequency-domain interval metrics consider the distribution of signal energy during a heartbeat (12, 13). Non-linear HRV metrics consider the complexity and unpredictability of the heartbeat (12). HRV can also be broken down and measured in different time frames: Ultra short term (<5 min), short term (~5 min) and long term (up to 24 h). Longer term measures of HRV have been more thoroughly implemented into health monitoring, however, short term measures have recently been proven to be just as effective. Despite some initial uncertainty regarding the time required to obtain an accurate reading for HRV measurements, numerous studies investigating this problem further have supported the idea that testing only requires a few minutes or less to produce accurate readings of some HRV metrics (14–16).

Although HRV monitoring is not very well integrated into the athletic community, the drastic increase in widely accessible and simple technology that can accurately test for this metric, such as smartwatches or other handheld devices (17–19), have made HRV more easily recorded and thus becoming a prominent physiological marker used to analyze various health conditions. Not only is this important to an athlete’s overall well-being, but studies have begun to draw a link between changes in HRV and head trauma, claiming that autonomic nervous system (ANS) irregularity is a common effect of head trauma in most instances. Despite some conflicting evidence regarding this finding (5, 6, 20), much of the research supports the idea that HRV and brain health go hand in hand. Given that heart rate variability responds to changes in the autonomic nervous system, it can often be used as a measure for ANS functionality. Therefore, decreased ANS from a TBI may be detectable through HRV measurements, making HRV a reliable test for TBI. Both a 2023 meta-analysis and randomized controlled trial investigated how HRV biofeedback treatment was able to improve brain function after a TBI. They found improvements in both emotional and cognitive function, including the reduction of symptoms such as headaches and sleep disturbances (21, 22). HRV can reveal that ANS is still unrecovered well beyond the recovery of symptoms, and taking this into account can prevent susceptible athletes from returning to play and risking repetitive head trauma before full recovery.

Despite evidence supporting the idea that ANS functionality can remain depressed long term after a concussion, few studies testing history of concussion can confirm a relationship between the two. Hence, our systematic review aims to answer whether HRV and ANS functionality at rest are altered when an athlete has a history of concussions. The scope of this study will focus on time-domain parameters, due to the accuracy of short term HRV tests on these measures. The goal is to add to the current pool of literature revolving HRV testing at rest and determine if it is a reliable way to test for cognitive deficits in athletes when deciding when they are ready to return to play.

## 2 Materials and methods

### 2.1 Search strategy

PubMed, Google Scholar, Cochrane Library, and EMBASE search engines were used to perform a systematic review using the PRISMA (Preferred Reporting Items for Systematic Reviews and Meta-Analyses) guidelines. These databases were searched by two authors (EW and ZA) for material dating back to 1996, when the Task Force of the European Society of Cardiology and the North American Society of Pacing and Electrophysiology first defined heart rate variability into its domains. All studies from then up to July 2023 were considered. Searches were conducted using a combination of various keywords, synonyms, headings (MeSH) and entry terms such as: “hrv” or “heart rate variability,” “concussion” or “sports concussion,” “tbi” or “traumatic brain injury,” and “history of concussion.”

### 2.2 Selection criteria

Only studies dealing with the impact of concussion history on HRV values at rest were considered for this meta-analysis. There was no limit on the time or location of publishing, or the types of studies conducted. Only studies in English were recovered. The eligibility criteria were: (1) studies including participants with an average age range of 13–40; (2) must include resting HRV values for both subjects with concussion history, and subjects without concussion history, in at least one time-domain parameter; and (3) Short term/ultra short term HRV must be measured with an electrocardiogram, however there was no specification as to what devices were allowed. Exclusion criteria were (1) studies involving subjects with a history of chronic health conditions; (2) studies done on those were had a history of moderate or severe TBI as defined by the Glasgow Coma Scale; and (3) participants have sustained a concussion within the past 4 months. The article searching process is shown below in accordance with the criteria. Four studies were excluded in the last process. These four studies fit the inclusion criteria; however, they were either unclear with data presentation, or they isolated the data too much with variables (gender, age) unlike the others.

### 2.3 Risk of bias assessment

A risk of bias assessment was conducted using the ROBINS-E tool. Risk of bias against the seven domains was assessed by two authors (EW and ZA) and any discrepancies were resolved through discussion.

### 2.4 Statistical analysis

A meta-analysis was conducted from at least three studies that reported data in a homogenous way. In the meta-analysis, the impact of history of concussion was assessed using an electrocardiogram to measure HRV values at rest for the time-domain parameters NN (RR; interval between successive heart beats), SDNN (standard deviation of the NN (RR)), and RMSSD (root mean square of successive RR interval differences). The difference in values and significance levels were then used to determine if there is a notable difference in HRV values for the two groups. We used Review Manager (RevMan 5.4, Cochrane Informatics & Technology, London, UK) to determine the Q and I^2^ statistics (in percentages) to establish variation between the studies attributed to heterogeneity. The meta-analysis was conducted using the continuous data function employing a random effects model and reporting mean difference. Where a meta-analysis was not possible, a narrative synthesis approach was used to analyze the data.

## 3 Results

### 3.1 Study selection

**Figure 1:**
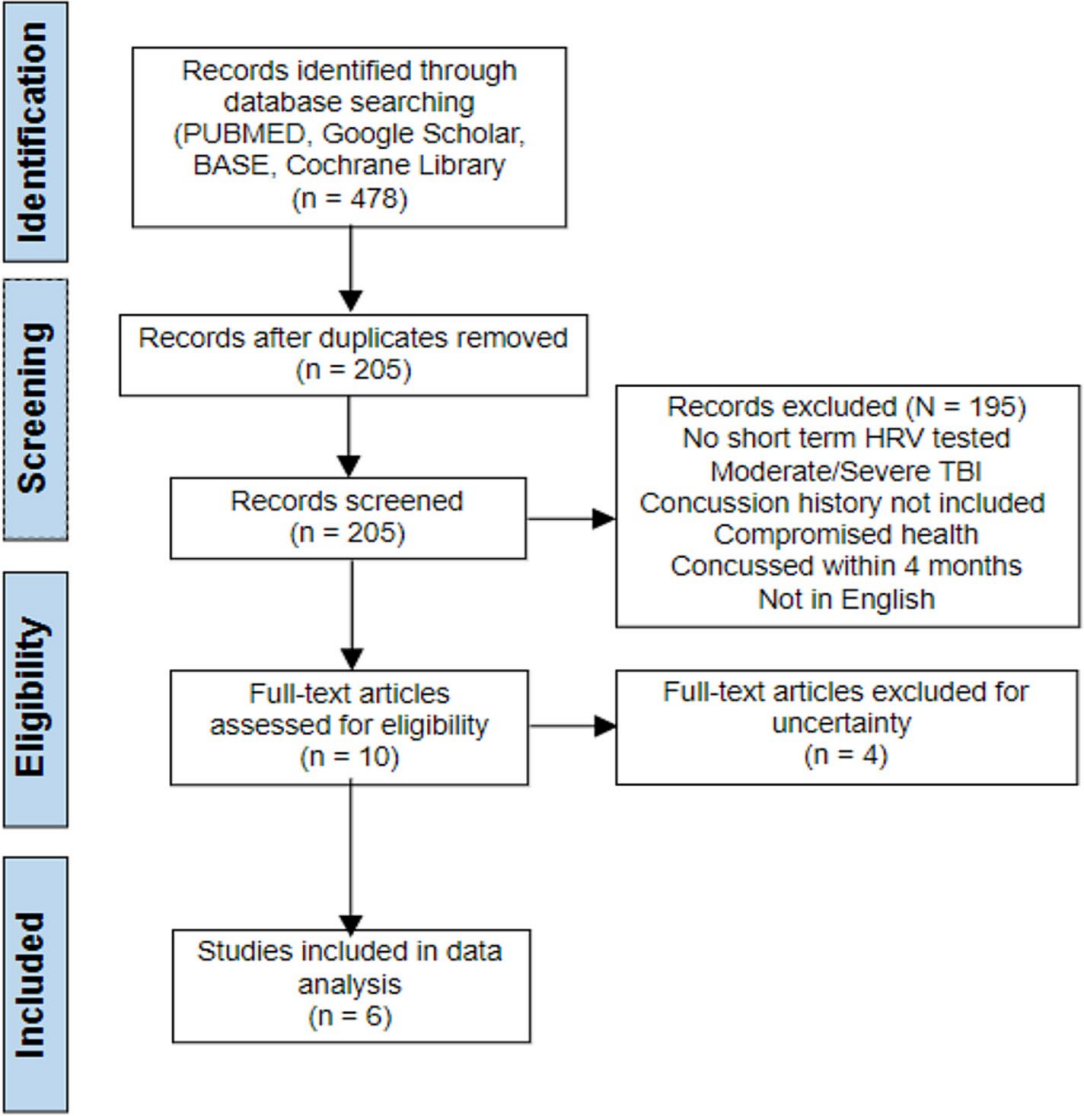
PRISMA flow chart.

The search strategy identified 478 articles through the data search. Only 205 records remained after duplicates were removed and 195 studies were excluded. After title and abstract screening, 10 studies remained from which four were excluded after full text reading, leaving six studies for the narrative synthesis (Figure 1).

### 3.2 Study characteristics

A summary of the study characteristics, including demographics, tested parameters, and general conclusions are listed below in Table 1. All the included studies were controlled trials however they were not randomized, due to the inability to randomly assign a history of concussion to a subject. Therefore, the testers knew who had a history of concussion and who did not.

**Table 1:** Characteristics of the studies.

Study	Subjects (n)	Mean age (years)	HRV metrics tested (in time-domain)	Findings (at rest)
Hilz (2016) (23)	Healthy-29 (9 women) TBI-25 (7 women)	Healthy- 31.3 ± 12.2 TBI-35 ± 13.2	MeanNN, SDNN, RMSSD	SDNN and RMSSD lower in history of concussion group (seated)
Hilz (2011) (24)	Healthy-20 TBI-20 (3 women)	Healthy-25.6 ± 8.8 TBI-37 ± 13.3	MeanNN, SDNN, RMSSD	MeanNN, SDNN and RMSSD lower in history of concussion (supine)
Memmini (2021) (25)	Healthy-18 (male) TBI-15 (male)	Healthy-16 ± 1 TBI-16 ± 1	SDNN, RMSSD	No significant difference in SDNN or RMSSD (seated)
Harrison (2022) (26)	Healthy-18 (male) TBI-16 (male)	Healthy-15.98 ± 0.62 TBI-16.06 ± 0.73	MeanNN, SDNN, RMSSD	No significant difference for any metric (seated)
Hilz (2015) (27)	Healthy-27 TBI-24 (7 women)	Healthy-30 ± 11 TBI-34 ± 12	MeanNN	No significant difference in MeanNN between groups (supine)
Haider (2020) (28)	Healthy-21 TBI-9 (5 female)	Healthy-18.3 ± 2.0 TBI-16.7 ± 3.0	MeanNN, RMSSD	No significant difference in either metric (supine)

There were a total of 242 subjects included in the studies; 109 subjects had a history of at least one concussion, while 133 subjects in the control group had no history of concussion. The average age of the control group was 23.3 ± 8.2, while the average age of the history of TBI group was 25.4 ± 9.7. There was no significant statistical difference between the two groups (*p*-value <0.202). Four of the six studies included females (23, 24, 27, 28) and none of them separated the values by gender. Only 31 of the total 242 subjects were confirmed to be female. Due to this inconsistency and lack of representation, this analysis was unable to determine differences in HRV on a gender basis. Half of the studies included all three tested metrics as outcomes measures, while the other half included either one or two of the tested metrics.

### 3.3 Risk of bias assessment

**Figure 2:**
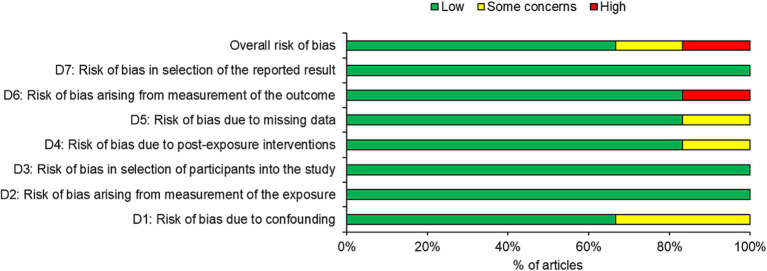
Risk of bias in the included studies.

Four of the six studies were judged as having low risk of bias with two studies judged as having some risk or high risk of bias (Figure 2). Overall, one third of the articles had some concerns or high risk of bias, with the high risk of bias occurring in domain 6 (risk of bias arising from measurement of the outcome). One third of the studies had a high risk of bias in domain 1 (risk of bias due to confounding). One study each also possessed some concerns in domain 4 and 5.

### 3.4 Results of studies

Only two of the studies found statistical significance at rest between those with a concussion history, and those without. Hilz et al. in their 2011 study (24) found a significant difference at rest for all three metrics: Mean NN (*p* < 0.006), SDNN (*p* < 0.005), and RMSSD (*p* < 0.043). In a similar study conducted 5 years later, Hilz et al. again demonstrated in their 2016 study (23) that when laying in a supine position, resting values for the history of concussion group were statistically significant in SDNN (*p* < 0.043), and RMSSD (0.005). The authors note that the Mean NN values for the history of concussion group were lower on average than the controls, however it never reached significance (*p* < 0.17). Neither of the other four studies found any notable difference at rest at *p* < 0.05 for any of the three metrics focused on in this analysis.

**Figure 3:**
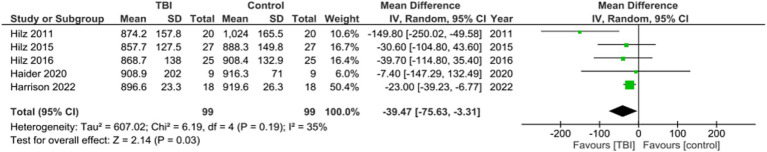
Meta-analysis for Mean NN between TBI and control groups.

Meta analysis for studies reporting Mean NN found the mean difference between the TBI and control groups favored TBI with a significant mean difference of −39.47, 95% CI [−75.63, −3.31], *p* = 0.03 (Figure 3). One of the studies did not include MeanNN as a metric (25). These values indicate that MeanNN at rest was significantly lower in those with a history of concussion compared to those without a history of concussion.

**Figure 4:**
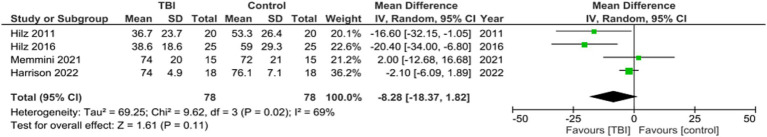
Meta-analysis for Mean SDNN between TBI and control groups.

Meta analysis for studies reporting data on Mean SDNN found the mean difference between the TBI and control groups was not significant, with a mean difference of −8.28, 95% CI [−18.37, 1.82], *p* = 0.11 (Figure 4). Two out of the six studies did not include SDNN as a metric (27, 28). These values indicate that the SDNN of the group with no history of concussion was not significantly different than the SDNN of the group with a history of concussion.

**Figure 5:**
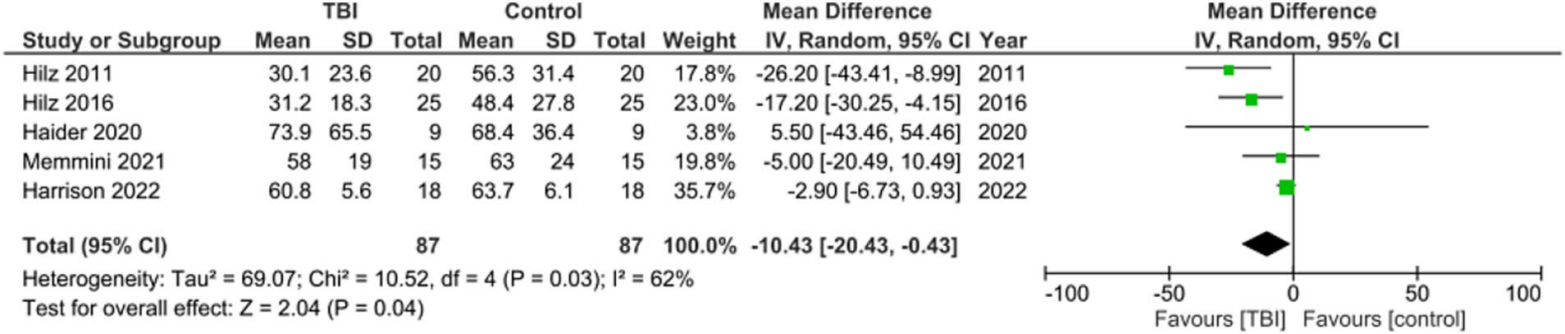
Meta-analysis for mean RMSSD between TBI and control groups.

Meta analysis for studies reporting data on Mean RMSSD found the mean difference between the two groups favored TBI with a significant mean difference of −10.43, 95% CI [−20.43, −0.43], *p* = 0.04. One out of the six studies did not include RMSSD as a metric (Figure 5) (27). These values indicate that the RMSSD of the group with no history of concussion was significantly higher than the RMSSD of the group with a history of concussion.

## 4 Discussion

This systematic review was conducted to decipher the impact that a history of concussion can have on resting HRV values. Short term and ultra short term HRV methods were utilized to measure resting HRV values. The three time-domain parameters of HRV included were MeanNN, SDNN, and RMSSD. After researching various databases, six total studies consisting of 242 subjects (109 HOC, 133 healthy) were analyzed. Based on the information collected, it was found that a history of concussion was a significant factor in influencing HRV values at rest for MeanNN and RMSSD, but not SDNN. While at rest, MeanNN and RMSSD were shown to present significantly different values between controls and history of concussion subjects, however SDNN did not reach significance. These results were closely in line with two of the six individual studies. Hilz et al. in 2011 found all three of the studied parameters to be statistically lowered in the history of concussion group. They write that the TBI patients had higher resting heart rate, yet a reduced ability to modulate their heart rate, shown in the significantly lowered HRV values (23, 24). However, the other four studies did not conclude a similar thing and were unable to find a find a difference in these parameters at rest.

RMSSD has been linked strongly to the variance in heart beats, and it helps estimate the changes in HRV caused by the vagus nerve (29). Due to this strong connection between RMSSD and the actions of the vagus nerve, Hilz writes that “These changes at rest imply a reduced ability of mTBI patients to modulate and buffer bursts of sympathetic activation” (24). The reduced overall HRV puts the post-TBI subjects at risk for sympathetic hyperactivity, further attributing the reduced HRV values to an imbalance between the sympathetic and parasympathetic systems. This finding was repeated in their 2016 study (23). The impact of concussion on the vagus nerve capabilities and sympatho-vagal processes to monitor the ANS long term has been also noted in other previous studies (30–32). Vagal functioning is also closely linked to other neurological conditions. Epilepsy, migraines, dementia, etc. have all been shown to worsen due to a lowered HRV. Concussion frequency and severity can influence the development of these conditions, and although the degree of HRV decreases can differ based on factors such as the severity of a TBI, the negative impact that a lower HRV has on health remains the same whether it is caused by a TBI or some other neurological disease (33). Despite the authors writing that concussion is tied to ANS deficits, the other studies in this meta-analysis found no difference in values at rest and conclude that only in the presence of stressors will the ANS and HRV deficits become known (27, 30, 34). Although not fully understood why this is the case, Gall et al. suggests that “the relatively mild neurological damage suffered as a result of a concussion may be insufficient to induce a detectable neuroautonomic cardiovascular dysfunction at rest” (34). This statement is supported by Katz-Leurer et al. who tested patients with history of severe TBI against healthy controls (35). Although it is beyond the scope of this analysis, researching literature involving moderate and severe TBI may further reveal a difference between a history of mild TBI and a history of more severe cases.

SDNN did not show a significant difference between the two groups with the data collected. This contradicts two of the studies conducted by Hilz et al. who found SDNN to be significantly lower in the history of concussion group compared to the healthy controls (23, 24). SDNN has been highly correlated with activation of both the parasympathetic and sympathetic nervous systems and although it can give accurate readings in short term tests, it is a stronger predictor of health when it comes to 24-h recordings (13). It is a better indicator of slower cardiovascular processes and how the body reacts to various stimuli. This may explain why no significant difference was found in the short-term measurements used in this analysis. It is important to touch on the sensitivity of HRV measurements because short term and ultra short term HRV metrics are not free of flaws and could be responsible for impacting the results. These quicker tests can be affected by transitional effects occurring with in the body (36), and some of the slower heart fluctuations cannot be read (19, 36, 37). Similarly, there is a difference regarding the position in which one is resting that can have an impact on the HRV measurements as well. Three of the studies specified that the resting position was supine (24, 27, 28). Hilz et al. showed significant differences between standing and sitting values in both MeanNN and SDNN despite both positions being in the resting state (24). This difference was shown in another study, when comparing sitting resting values to standing resting values (37). These slight changes in the orientation of the body can change the load on the cardiovascular system, which can impact HRV measurements drastically. Pairing that with a more sensitive metric such as SDNN, it may explain the lack of significance in resting SDNN values between the groups.

One thing that was not specified in the inclusion criteria for this analysis was the number of prior concussions in the TBI groups. Although three of the studies included the number of prior concussions in the demographics (25, 26, 28), only one of the included studies conducted an analysis to stratify the groups by number of prior concussions, to find out if the deficits to HRV last longer or are more prominent as the number of prior concussions increases. They conclude that those with a history of two or more concussion may be susceptible to long-term or even permanent alterations in ANS neurotransmission, and that a history of multiple concussions may negatively impact recovery of the ANS after a bout of exercise (25). Contrarily, this is not shown to be the case in other studies that found no significant difference (38). Furthermore, mechanisms of the HRV changes were neither included in the criteria, nor specifically noted in the studies. HRV can be impacted by things such as inflammation, with the increase of pro-inflammatory cytokines presenting a negative association with HRV (39). Similarly, intracranial pressure and its impact on perfusion and blood flow play a role as well (11).

Due to cardiovascular processes increasing with exercise, another prominent issue is the ability to measure HRV during these times, with some researchers questioning the accuracy of short-term readings during physical activity (15, 16, 40). When measuring for altered HRV, differences are potentially much more present during activity compared to rest and may not be detectable at all without some form of exercise (5, 23, 30). Exercise HRV has been shown to increase during exercise in all groups. However, individual differences in HRV parameters between concussed and non-concussed groups may be more prominent during exercise when compared to rest. La Fountaine found lowered HRV levels during the acute stage in concussed individuals during a handgrip exercise that was not found during rest (41). Abaji et al. followed a similar exercise protocol and found that during physical exertion, concussed patients had a reduced HRV response in both time and frequency-domain parameters, even in the post-acute stage after symptoms have gone (30). A more demanding cycle ergometer test was used to investigate this idea by Gall et al. (34) to see if the results held true. They found no difference in resting values in any parameter of HRV, however, concussed subjects demonstrated lower MeanNN and other frequency parameters during the cycle exercise protocol. Touched on by some of these studies, the differences found through these experiments may not be completely true. Some compounding factors such as detraining effects from taking time off to heal from a concussion, or differing fitness levels between the concussed subjects and their matched healthy controls could account for some of the HRV differences (24, 25, 28). To further prove this concept, a more consistent exercise protocol amongst studies would be crucial. That was not the case for this analysis, so comparing them based on exercise HRV values would have been inappropriate.

Although this analysis focused on the longer-term effect of a history of concussion on autonomic nervous system functionality, there is stronger evidence to suggest that concussion has greater effects on the ANS in the more acute stages. Ellingson et al. found SDNN to be depressed after a 5-min ECG recording in collegiate aged athletes 1–5 days post-concussion (42). Another study on Canadian hockey players found that while standing at rest, the concussed group had lower SDNN and nearly significant lowered RMSSD (31). Two similar studies investigated differences in HRV parameters immediately after concussion, during exercise while asymptomatic, and 1-week post return to play and compared the results to healthy matched controls. One study found the concussed group to be lower in various frequency parameters, and that history of concussion may play a role in this (43). The other also found significant differences in some frequency measures, however also demonstrated lowered MeanNN and SDNN in concussed individuals (44). Regardless of the individual findings, both studies confidently concluded that the resolution of symptoms does not reflect recovery of the ANS, and that significant HRV abnormalities can remain even after return to play. In other forms of TBI, there may be additional lasting consequences to ANS functionality, such as neural damage from brain hemorrhage or torn axons.

## 5 Limitations

This systematic review has several limitations. One such limitation is the lack of homogeneity of the studies regarding gender inclusion. Although four of the six studies included women (23, 24, 27, 28), none separated males and females into their own groups. This introduces some uncertainty in the results, as gender has been previously shown to occasionally influence HRV values at rest (36, 45, 46). Despite the results not always being significant, it is still important to consider but was not implemented into this analysis. Age can play a similar role in influencing HRV, with elderly populations proven to have a reduced HRV, due to the lowered health status that comes with aging (6, 21, 24). The included studies did combat this potential factor by ensuring there was no significant age difference between the TBI and control groups, so any underlying influence that age would have on the experiment would be evenly distributed between the two groups. When it comes to the age difference between studies, one study had an age range of 18–59 (24), while others focused on young male athletes aged around 16 (25, 26). Finding a more homogenous population in age and gender would help minimize any such differences.

When considering the history of concussion, some of the information in these studies was self-reported. This creates uncertainty regarding the truth to the number of reported concussions, along with recall bias as to how long ago the concussions occurred. Another limitation in most HRV studies is the lack of a baseline test prior to head trauma. Although studies use healthy, age and sex matched controls to make HRV values as comparable as possible, there is a level of assumption that is unable to account for the individuality in HRV values from one person to the next. Fitness levels can play a role in altering HRV (47–49), and genetic factors can also impact these values slightly (36, 50). Other conditional variables include anxiety levels, stress, and emotional states (21, 51, 52). Future studies regarding HRV should start with baseline tests for all athletes in the subject population, allowing comparisons to be made individually, to better display how a history of concussions can affect HRV values.

## 6 Conclusion

In conclusion, this meta-analysis investigated if a prior history of concussion had a lasting long-term effect on HRV values. The implications of the material were to further investigate whether HRV can be used to test ANS functionality and be used on athletes to determine if they are able to return to play. Resting HRV values in the time-domain were researched for the metrics of MeanNN, SDNN, and RMSSD. Six studies were included, and the results showed a significant difference in resting values between the two groups in MeanNN and RMSSD. History of concussion may lower HRV at rest, therefore it is important to consider when evaluating a concussed athlete. History of concussion may hint at an increasingly compromised ANS and could help prevent a vulnerable athlete from returning to play prematurely. Factors such as age, gender, or time since last concussion could have played a role in influencing results. Inconsistencies between studies lingered, so future studies should find a standardized procedure and more homogenous study group to confirm any relationship.

## Data availability statement

The original contributions presented in the study are included in the article/supplementary material, further inquiries can be directed to the corresponding author.

## Author contributions

EW: Conceptualization, Data curation, Formal analysis, Investigation, Methodology, Writing – original draft. ZA: Conceptualization, Data curation, Formal analysis, Methodology, Supervision, Writing – review & editing. VP: Conceptualization, Data curation, Methodology, Supervision, Writing – review & editing.
